# DNA repair protein Rad18 restricts LINE-1 mobility

**DOI:** 10.1038/s41598-018-34288-9

**Published:** 2018-10-26

**Authors:** Yasuo Ariumi, Koudai Kawano, Mariko Yasuda-Inoue, Misao Kuroki, Hiroyuki Fukuda, Rokeya Siddiqui, Priscilla Turelli, Satoshi Tateishi

**Affiliations:** 1 Center for AIDS Research, Kumamoto University, 2-2-1 Honjo, Chuo-ku, Kumamoto, 860-0811 Japan; 2 School of Life Science, Ecole Polytechnique Fédérale de Lausanne, Lausanne, CH-1015 Switzerland; 3 Institute of Molecular Embryology and Genetics, Kumamoto University, 2-2-1 Honjo, Chuo-ku, Kumamoto, 860-0811 Japan

## Abstract

Long interspersed element-1 (LINE-1, L1) is a mobile genetic element comprising about 17% of the human genome. L1 utilizes an endonuclease to insert L1 cDNA into the target genomic DNA, which induces double-strand DNA breaks in the human genome and activates the DNA damage signaling pathway, resulting in the recruitment of DNA-repair proteins. This may facilitate or protect L1 integration into the human genome. Therefore, the host DNA repair machinery has pivotal roles in L1 mobility. In this study, we have, for the first time, demonstrated that the DNA repair protein, Rad18, restricts L1 mobility. Notably, overexpression of Rad18 strongly suppressed L1 retrotransposition as well as L1-mediated Alu retrotransposition. In contrast, L1 retrotransposition was enhanced in Rad18-deficient or knockdown cells. Furthermore, the Rad6 (E2 ubiquitin-conjugated enzyme)-binding domain, but not the Polη-binding domain, was required for the inhibition of L1 retrotransposition, suggesting that the E3 ubiquitin ligase activity of Rad18 is important in regulating L1 mobility. Accordingly, wild-type, but not the mutant Rad18-lacking Rad6-binding domain, bound with L1 ORF1p and sequestered with L1 ORF1p into the Rad18-nuclear foci. Altogether, Rad18 restricts L1 and Alu retrotransposition as a guardian of the human genome against endogenous retroelements.

## Introduction

Long interspersed element type 1 (LINE-1, L1) is an active and autonomous non-long terminal repeat (LTR) retrotransposon composed of approximately 17% of the human genome^1–5^. L1 encodes two proteins, ORF1p with RNA-binding and nucleic acid chaperone activities and ORF2p with endonuclease and reverse transcriptase activities required for L1 retrotransposition^1,3–7^. ORF1p and ORF2p assemble with L1 mRNA and form a ribonucleoprotein (RNP) in the cytoplasmic foci^8,9^. L1 propagates by a target primed reverse transcription (TPRT) after the L1-RNP complex enters the nucleus. The L1 endonuclease creates a nicked DNA that serves as a primer for reverse transcription of the L1 RNA, leading to integration of L1 cDNA into the human genome^10,11^. The typical L1 endonuclease cleavage site is 5′-TTTT/AA-3′^10–12^. Thus, L1 insertion generates DNA double-strand breaks (DSBs) by L1 endonuclease in the target DNA^13^.

The ataxia-telangiectasia mutated (ATM) is activated by DSBs and subsequently phosphorylates downstream substrates, including p53, Chk2, BRCA1 and MRE11-Rad50-NBS1 complex, resulting in the activation of the DNA damage checkpoint and cell cycle arrest^14^. Accordingly, L1 retrotransposition was increased in ATM-deficient cells, indicating that the ATM signaling pathway suppresses L1 retrotransposition^15^. Thus, the DNA damage response may modulate L1 mobility. Furthermore, host DNA repair machinery may also affect L1 retrotransposition. In fact, deficiencies of the non-homologous end-joining (NHEJ) repair pathway such as Ku70 and DNA ligase IV decrease L1 retrotransposition, suggesting that NHEJ repair pathway is required for efficient L1 retrotransposition^16^. In contrast, Morrish *et al*. showed that L1 can retrotranspose efficiently in NHEJ-deficient Chinese hamster ovary (CHO) cells^17,18^. In addition, the DNA repair enzyme ERCC1/XPF complex inhibits L1 retrotransposition^19^.

Although DNA lesions caused by UV light or chemical mutagens are efficiently removed and repaired by nucleotide excision repair, DNA damage still remains during DNA synthesis and causes DNA replication to stall. To avoid this stalling, post-replication repair protein Rad18 contributes to damage bypass and DNA repair. Rad18 is an E3 ubiquitin ligase recruited to the DNA damage site. The DNA replication fork stalled by DNA damage induces monoubiquitination of DNA polymerase adaptor, proliferating cell nuclear antigen (PCNA), by the Rad6/Rad18 ubiquitin ligase complex^20–26^. The PCNA monoubiquitination triggers the replacement of replicative DNA polymerase δ with translesion synthesis polymerase η that can replicate DNA lesions^25,26^. Interestingly, PCNA interacts with ORF2p via a PIP box motif ^27^. Importantly, this interaction is critical for L1 retrotransposition^27^. Rad18 deficiency causes gross chromosomal rearrangement in yeast^28^ and genomic instability, and an increase of sister chromatid exchange in chicken and mouse cells^29,30^. Furthermore, Rad18 is known to interact with human immunodeficiency virus type 1 (HIV-1) integrase^31^, which catalyzes the HIV-1 integration process, and suppresses the early step of HIV-1 infection^32^, suggesting a role of DNA repair-mediated genomic stability maintenance in viral infection. In this study, we examined the effect of Rad18 on the L1 mobility.

## Results

### Rad18 restricts L1 retrotransposition

**Figure 1:**
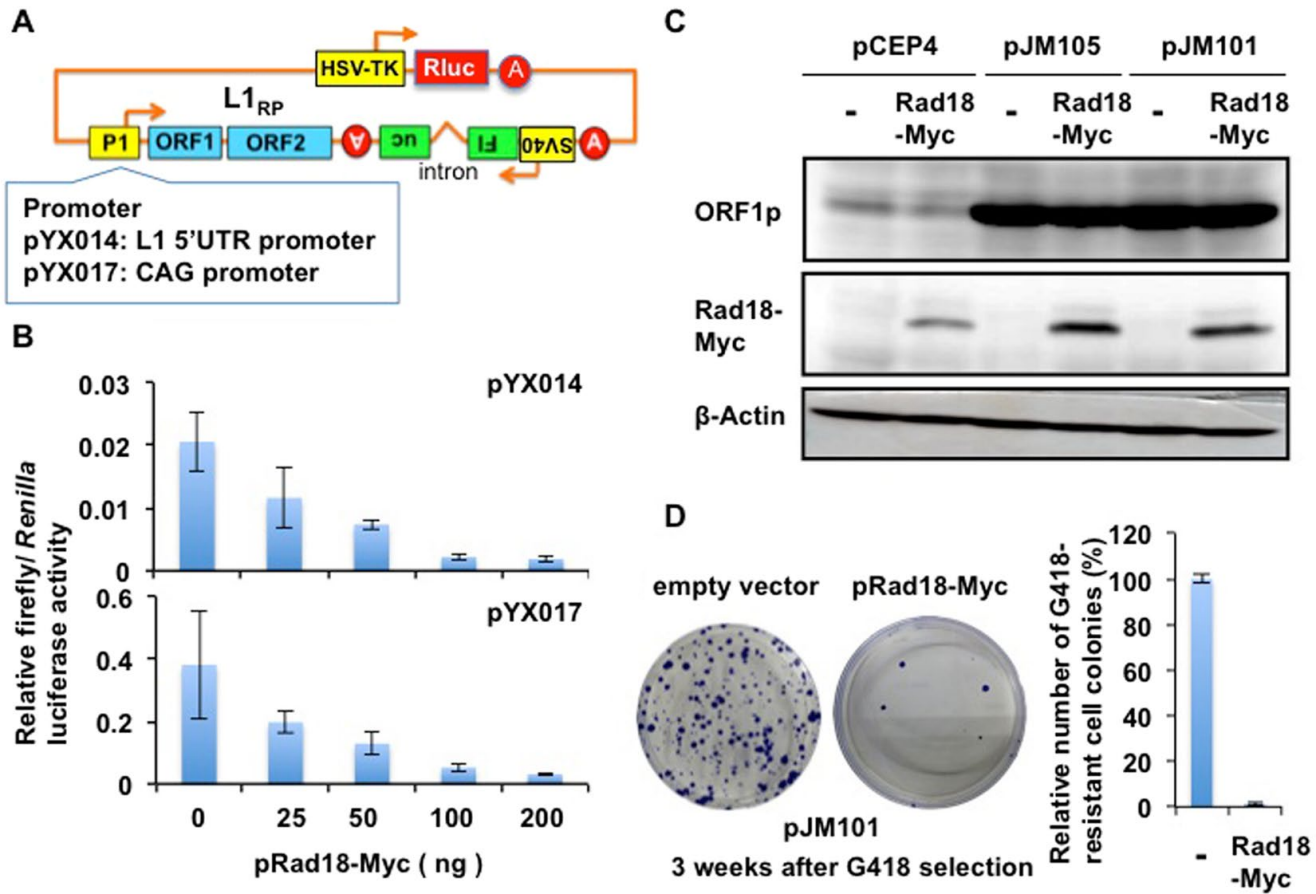
Rad18 suppresses L1 retrotransposition. (**A**) Schematic representation of the firefly luciferase-based retrotransposition reporter cassette (pYX014 and pYX017)^33^. The pYX014 plasmid is a dual-luciferase reporter, in which firefly luciferase is used as the retrotransposition indicator and *Renilla* luciferase is encoded on the same plasmid for normalization. The L1_RP_ 5′UTR (pYX014) promoter was replaced by a strong CAG promoter and generated pYX017. (**B**) 293T cells (2 × 10^4^ cells/well) were co-transfected with Myc-tagged Rad18-expressing plasmid^22^ at the indicated amounts with either pYX014 or pYX017 (100 ng). Luciferase assays were performed three days after transfection in three independent experiments. Graph shows the mean (±SEM) firefly luciferase activity normalized with *Renilla* luciferase activity. (**C**) Protein expression level of L1 ORF1p in presence of Rad18. 293T cells (2 × 10^5^ cells/well) were cotransfected with 2 μg of pCEP-GFP, pJM105/L1.3 reverse transcriptase-deficient mutant^35–37^, or pJM101/L1.3 wild-type L1^35–37^, and 2 μg of pcDNA3-HA, or pRad18-Myc. Cells were cultured for 3 days, lysed, and subjected to Western blot to analyze the expression of ORF1p using anti-hORF1P antibody (SE-6798)^34^. Western blotting of the cell lysates with anti-ORF1p, anti-Myc-tag, and anti-*ß*-actin antibodies is also shown, respectively. (**D**) HeLa cells (2 × 10^5^ cells) were co-transfected with 2 µg of pJM101^35–37^ and 2 µg of pRad18-Myc^22^. Colony formation assays performed three weeks after G418 selection. G418-resistant colonies were stained with Coomassie brilliant blue. The Number of colonies was counted by Eliphoto counter (Minerva Tech, Tokyo, Japan). Graph shows the mean (±SEM) number of G418-resistant cell colonies with the condition without Rad18-Myc set to 100%.

**Figure 2:**
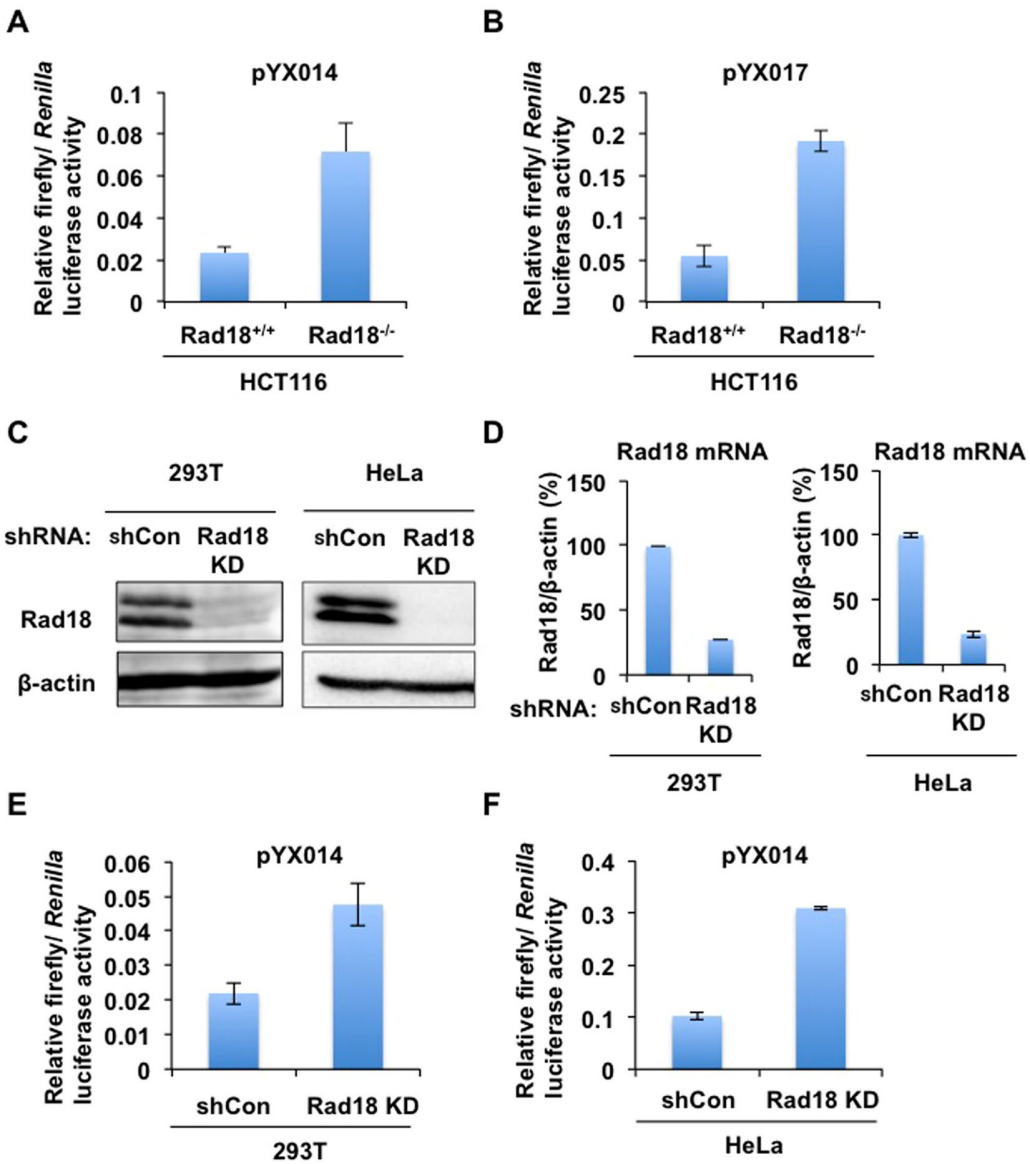
L1 retrotransposition in Rad18-deficient HCT116 cells or Rad18-knockdown 293T cells. (**A**, **B**) Rad18^+/+^ or Rad18^−/−^ HCT116 cells (2 × 10^4^ cells/well) were transfected with either pYX014 (**A**) or pYX017 (**B**) (100 ng). The luciferase assay was performed three days after transfection in three independent experiments. Graph shows the mean (±SEM) firefly luciferase activity normalized with *Renilla* luciferase activity. (**C**) Inhibition of Rad18 protein expression by shRNA-producing lentiviral vector. The results of Western blot analysis of cellular lysates with anti-Rad18 or anti-*ß*-actin antibody in 293T or HeLa cells expressing shRNA targeted to Rad18 (Rad18KD) as well as in 293T cells transduced with a control lentiviral vector (shCon) are shown. (**D**) The levels of Rad18 mRNA in Rad18-knockdown 293T or HeLa cells (Rad18KD) or the control cells (shCon) were monitored by real-time LightCycler PCR, respectively. Experiments were done in triplicate, and graph shows the mean percentages (±SEM) of Rad18 mRNA normalized with *ß*-actin mRNA in the control cells set at 100%. (**E,F**) Rad18-knockdown 293T (**E**) or HeLa cells (Rad18 KD) (**F**) or the control cells (shCon) (2 × 10^4^ cells/well) were transfected with pYX014 (100 ng). Luciferase assays were performed three days after transfection in three independent experiments. Graph shows the mean (±SEM) firefly luciferase activity normalized with *Renilla* luciferase activity.

To investigate a potential role of DNA repair protein Rad18 in L1 retrotransposition, Rad18-expressing plasmids^22^ were co-transfected with pYX014 plasmid, which has the firefly luciferase-based retrotransposition reporter cassette^33^ (Fig. 1A) into 293T cells. pYX014 harbors the L1_RP_ 5′untranslated region (UTR) promoter as well as the firefly luciferase gene inserted in the 3′UTR of L1 in the opposite direction of L1 coding sequence^33^. The luciferase activity could be detected only after L1 retrotransposition has happened. Notably, Rad18 could strongly suppress the luciferase-based L1 retrotransposition activity in a dose-dependent manner (Fig. 1B). Substitution of the L1_RP_ 5′UTR promoter (pYX014) by a strong CAG promoter resulted in an increase of the retrotransposition activity (pYX017)^33^ (Fig. 1A,B). However, Rad18 also strongly suppressed the retrotransposition activity (pYX017) (Fig. 1A), suggesting that this inhibitory effect on L1 retrotransposition is independent of the L1 5′UTR promoter activity. To test whether Rad18 affects L1 transcription and L1-encoded protein expression, we used an anti-human ORF1 antibody (SE-6798)^34^. Rad18 did not alter endogenous ORF1p expression in 293T cells (Fig. 1C). Similarly, Rad18 did not suppress ORF1p when expressed from plasmids pJM101/L1.3 and pJM105/L1.3, which respectively contain WT and ORF2p RT-mutant (D702A) human L1.3 element in a pCEP4 backbone vector^35–37^ (Fig. 1C). Thus, Rad18 did not affect L1 transcription and L1 ORF1p expression. To further confirm this restriction, we examined G418-resistant colony assay using pJM101 harboring a neomycin resistance gene inserted in the 3′UTR of L1 in the opposite direction of L1 coding sequence as the selection marker^35–37^. Therefore, the number of G418-resistant cell colonies reflects the events of L1 retrotransposition. Consequently, Rad18 strongly reduced the number of G418-resistant cell colonies (Fig. 1D), indicating that Rad18 restricts L1 retrotransposition. Importantly, Rad18 did not affect cell viability (Fig. S1). However, we observed relatively small decreases (~10-fold reduction) in luciferase activity Fig. 1B when compared to the results of G418-resistant cell colony numbers (~100-fold reduction) in Fig. 1D. The differences in retrotransposition efficiencies may be associated with input amounts of Myc-tagged Rad18-expressing plasmids (~200 ng for luciferase assay; 2 µg for G418-resistant cell colony assay) even though Rad18 did not affect cell viability at 16 days post-transfection (Fig. S1). In contrast, L1 retrotransposition efficiency was elevated in Rad18-knockout (Rad18^−/−^) HCT116 human colon cancer cells^38^ compared to Rad18 wild-type (Rad18^+/+^) HCT116 cells (Figs 2A,B and S2A). Additionally, L1 retrotransposition was also enhanced in Rad18-knockdown 293T or HeLa cells (Fig. 2C–F). Thus, Rad18 seems to restrict L1 retrotransposition.

### Rad18 restricts L1-mediated Alu retrotransposition

**Figure 3:**
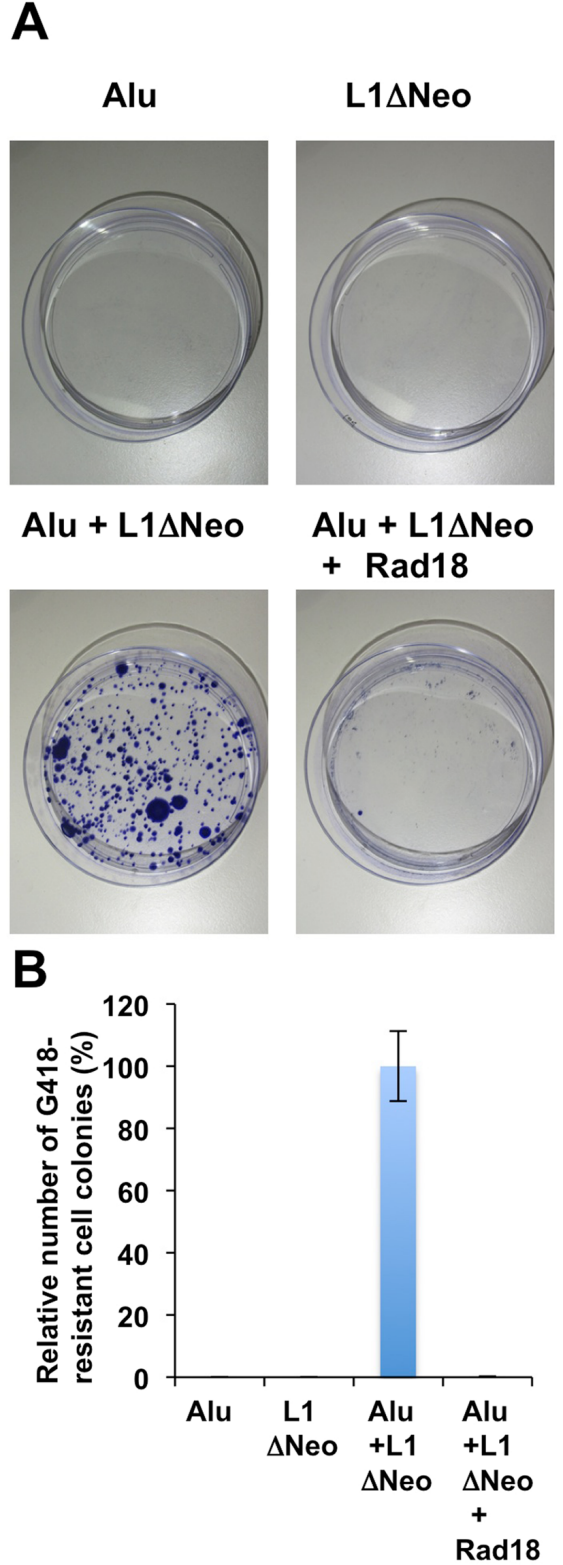
Rad18 suppresses L1-mediated Alu retrotransposition. (**A**) HeLa cells (2 × 10^5^ cells) were co-transfected with 2 µg of pAlu pA + neoTET^39^, 2 µg of pJM101 L1-RP∆Neo^36,41^ and/or 2 µg of pRad18-Myc^22^ and colony formation assays were performed four weeks after G418 selection. G418-resistant colonies were stained with Coomassie brilliant blue. (**B**) The Number of colonies was counted by Eliphoto counter (Minerva Tech, Tokyo, Japan). Graph shows the mean (±SEM) number of G418-resistant cell colonies when pAlu pA + neoTET and pJM101 L1-RP ΔNeo were co-transfected set to 100%.

Since L1 provides the *trans*-acting function required for the retrotransposition of non-autonomous retrotransposons such as short interspersed element (SINE), which includes Alu repeats in humans and SINE-VNTR-Alu (SVA)^39,40^, we utilized the G418-resistant cell colony assay to determine whether Rad18 could suppress L1-mediated Alu retrotransposition. When pAlu pA + neoTET^39^ and pJM101 L1-RP ΔNeo without the neomycin-resistant gene^36,41^ were cotransfected into 293T cells, L1 cooperated with Alu to form G418-resistant colonies, four weeks after G418 selection (Fig. 3A,B). However, we observed no G418-resistant colonies when either pA + neoTET or pJM101 L1-RP∆Neo alone was transfected. As expected, Rad18 markedly reduced the number of G418-resistant colonies, when pAlu pA + neoTET and pJM101 L1-RP∆Neo were cotransfected with Rad18-expressing plasmid. Importantly, Rad18 did not affect cell growth and cell viability (Fig. S1). These results suggested that Rad18 suppresses L1-mediated Alu retrotransposition (Fig. 3A,B).

### The Rad6-binding domain is important for L1 inhibition

**Figure 4:**
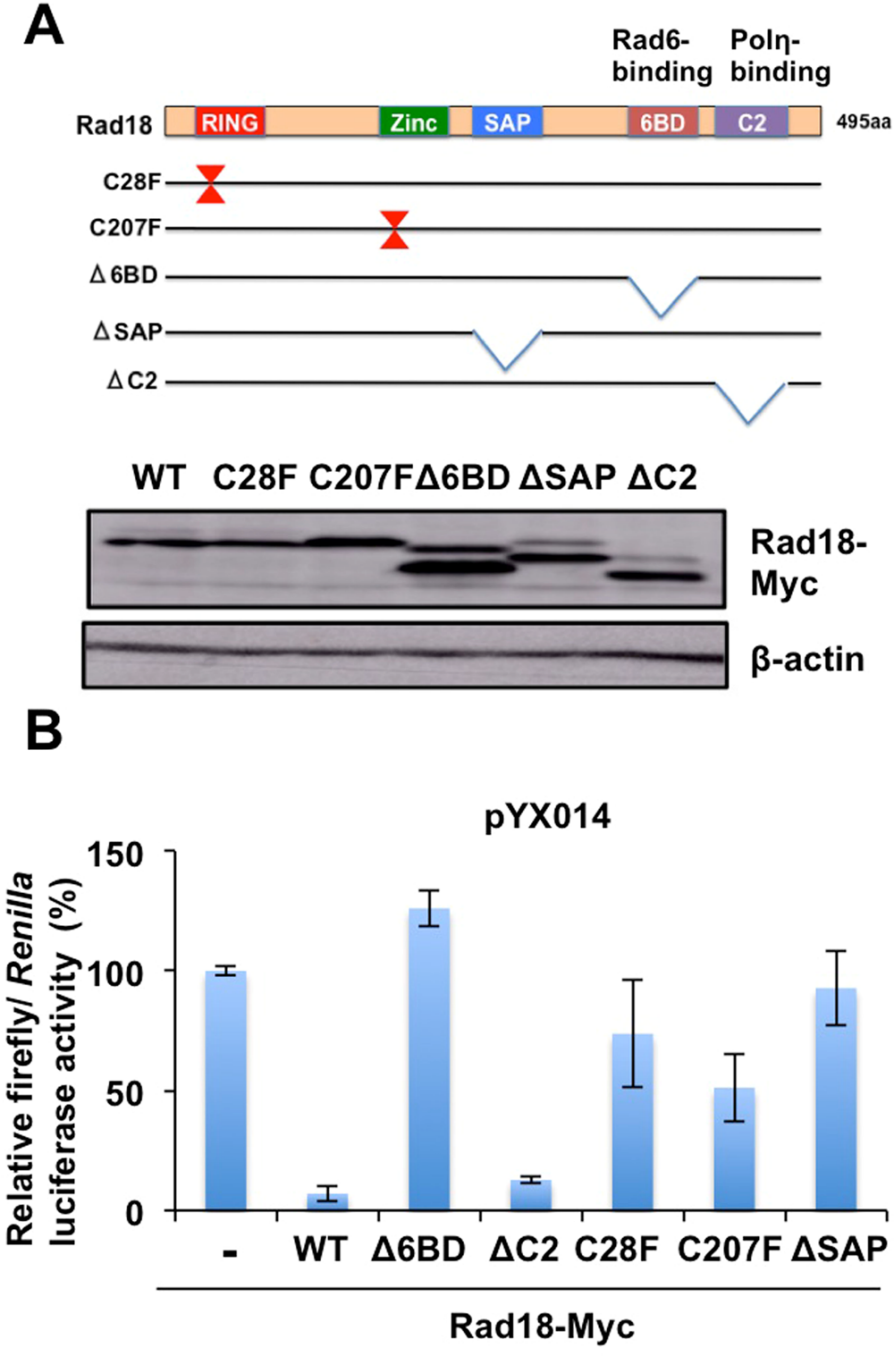
Functional Rad18 domains are required for L1 retrotransposition suppression. (**A**) Schematic representation of Rad18 and the mutants used in the present study. The RING-finger, Zinc-finger, SAP, Rad6-binding (6BD), and C2 domain are indicated. 293T cells were transfected with 2 µg of Myc-tagged Rad18 (WT, C28F, C207F, ∆6BD, ∆SAP, or ∆C2)-expressing plasmid^22^. Protein expression levels of Rad18 mutants, as well as WT Rad18, are shown by Western blot analysis with anti-Myc -tag or anti-*ß*-actin antibodies. (**B**) 293T cells (2 × 10^4^ cells/well) were co-transfected with 100 ng of Myc-tagged Rad18 (WT, C28F, C207F, ∆6BD, ∆SAP, or ∆C2)-expressing plasmid and 100 ng of pYX014. Luciferase assays were performed three days after transfection in three independent experiments. Graph shows the mean (±SEM) firefly luciferase activity normalized with *Renilla* luciferase activity with the condition without Rad18-Myc set to 100%.

Rad18 is an E3 ubiquitin ligase with multiple functional domains (Fig. 4A). The N-terminal RING-finger domain (residues 25–63) commonly found in E3 ubiquitin ligases is required for interaction with an E2 ubiquitin ligase, Rad6. In addition to the RING-finger domain, the C-terminal domain (6BD) (residues 340–395) is also required for Rad6 binding. A C2HC Zinc-finger domain (residues 201–225) is important for DNA binding. A SAF-A/B, Acinus and Pias (SAP) domain (residues 248–282) is also required for DNA binding. The C-terminal C2 domain (residues 401–445) is important for Polη binding^26^. To determine which Rad18 functional domain is required for inhibition of L1 retrotransposition, we used several Rad18 mutant-expressing plasmids^22^ (Fig. 4A). pYX014 and each Rad18 mutant-expressing plasmid were co-transfected into 293T cells. Consequently, the ∆C2 mutant without DNA Polη-binding domain could strongly suppress L1 retrotransposition like wild-type (WT) Rad18 (Fig. 4B). In addition, the RING-finger (C28F) and the ZINC-finger (C207F) domain mutants also suppressed L1 retrotransposition, however, the inhibition was relatively mild compared to the WT and the ∆C2 mutant (Fig. 4B). In contrast, the ∆6BD mutant lacking the Rad6-binding domain, and the ∆SAP mutant lacking the DNA-binding domain, failed to suppress L1 retrotransposition (Fig. 4B), even though we confirmed similar expression levels of these mutants compared to the WT Rad18 by Western blotting (Fig. 4A). Thus, both Rad6-binding domain and SAP domain are important for suppression of L1 retrotransposition, suggesting that the ubiquitin ligase activity of Rad18 is required for regulation of L1 mobility.

### L1 ORF1p localizes to P-bodies and stress granules

**Figure 5:**
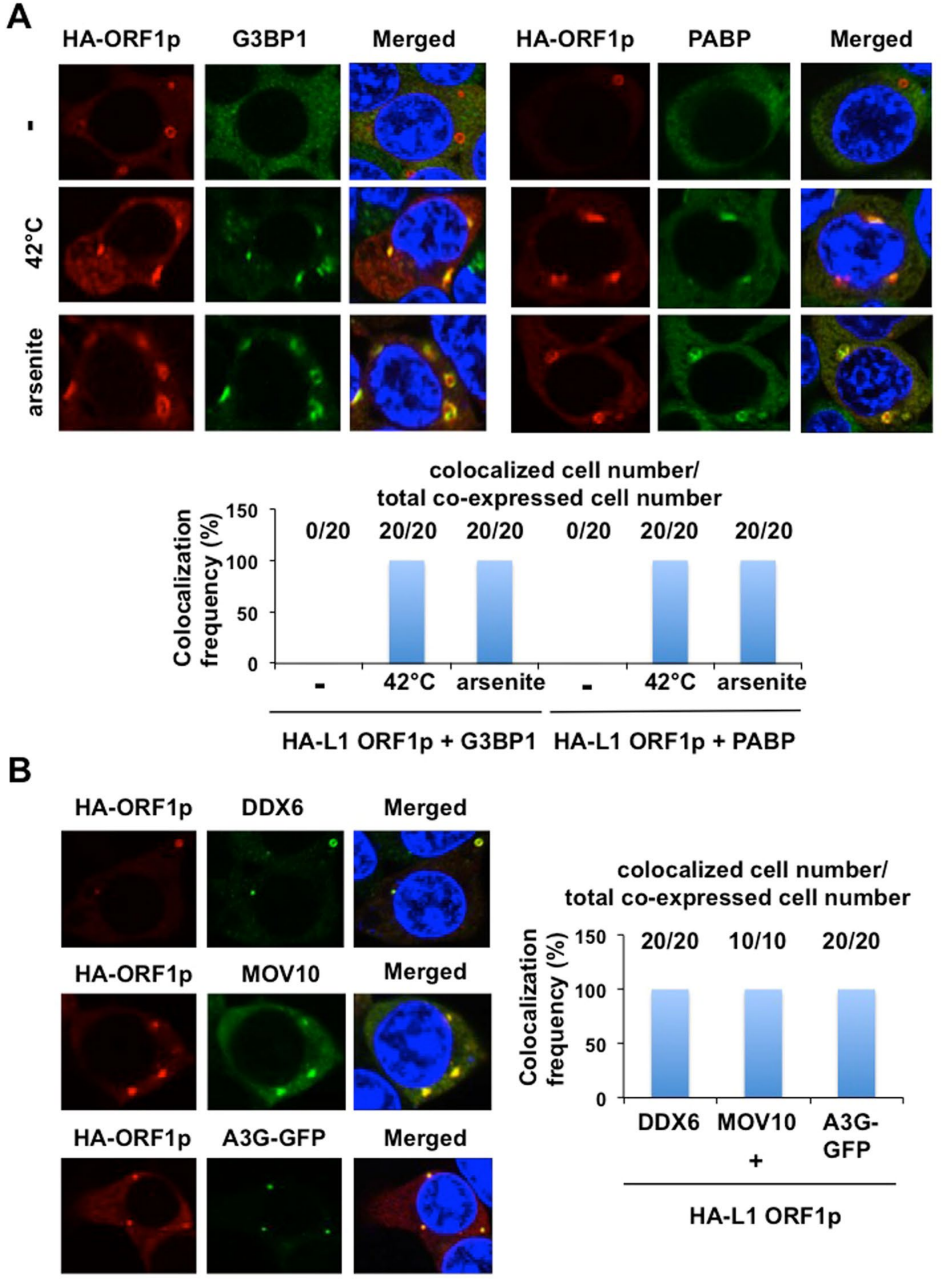
L1 ORF1p localizes to P-bodies and stress granules. (**A**) 293T cells (2 × 10^4^ cells/well) transfected with 200 ng of pcDNA3-HA-L1 ORF1 were incubated at 37 °C or 42 °C for 45 min. Cells were also treated with 0.5 mM arsenite for 30 min. Cells were stained with anti-HA (HA-7) and either anti-G3BP1 or anti-PABP antibodies and then visualized with Alexa Fluor 594 (HA-L1 ORF1p) or Alexa Fluor 488 (endogenous G3BP1 or PABP). Cells were examined by confocal laser scanning microscopy. Nuclei were stained with DAPI. The colocalization frequency is shown. We observed twenty co-expressed cells. (**B**) 293T cells (2 × 10^4^ cells/well) cotransfected with 200 ng of pcDNA3-HA-L1 ORF1, pcDNA3-FLAG-MOV10, or pA3G-GFP were examined by confocal laser scanning microscopy. Cells were stained with anti-HA (HA-7 or 3F10) and anti-DDX6, anti-FLAG (M2) antibodies and then visualized with Alexa Fluor 594 (L1 ORF1p), Alexa Fluor 488 (endogenous DDX6, MOV10) or EGFP (A3G-GFP). The colocalization frequency is shown. We observed twenty or ten co-expressed cells.

Although G3BP1 and poly(A)-binding protein (PABP), well-known stress granule components, were dispersed in the cytoplasm at 37 °C, both proteins formed discrete aggregates termed stress granules in response to heat shock at 42 °C for 45 min or treatment with arsenite for 30 min (Fig. 5A)^42^. We observed that L1 ORF1p dose not colocalize with G3BP1 or PABP at 37 °C, while L1 ORF1p colocalized with both G3BP1 and PABP in stress granules in response to heat shock at 42 °C (Fig. 5B). On the other hand, L1 ORF1p colocalized with DDX6, Moloney leukemia virus 10 (MOV10) and apolipoprotein B mRNA editing enzyme catalytic polypeptide-like 3 G (APOBEC3G, A3G), well-known P-body components, in P-bodies (Fig. 5B). Thus, L1 ORF1p seems to localize to P-bodies and stress granules.

### Rad18 interacts with L1 ORF1p

**Figure 6:**
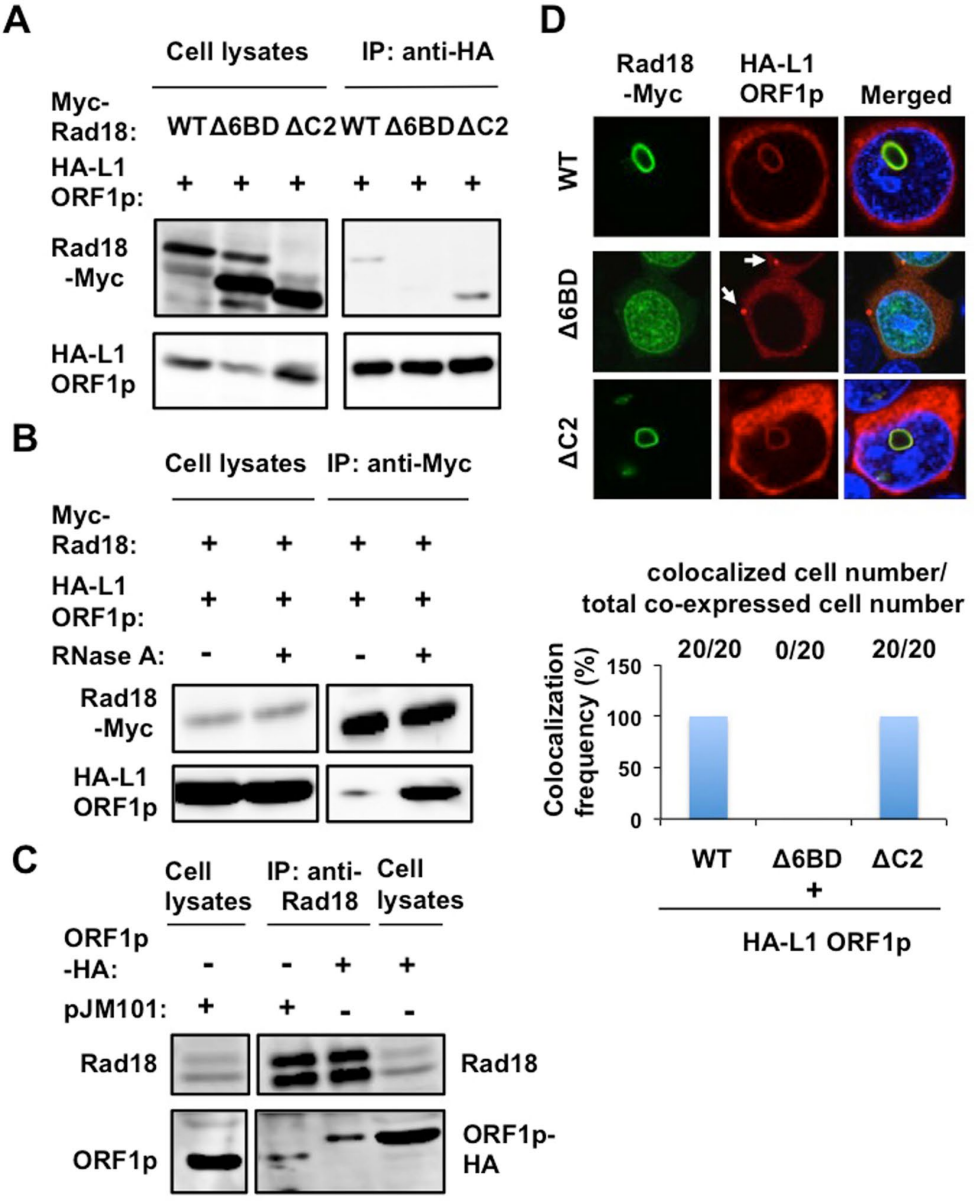
Rad18 interacts with L1 ORF1p. (**A**) 293T cells (2 × 10^5^ cells/well) were cotransfected with 2 µg of Myc-tagged Rad18 (WT, ∆6BD, or ∆C2)-expressing plasmid and 2 µg of pcDNA3-HA-L1 ORF1. The cell lysates were immunoprecipitated with anti-HA (3F10) antibody, followed by immunoblotting analysis using either anti-Myc-tag or anti-HA antibodies. The results of Western blot analysis of 1/10 of the cellular lysates with anti-HA or anti-Myc-tag antibodies are also shown. The experiment was replicated twice with similar results. (**B**) RNA-independent interaction of L1 ORF1p with Rad18. 293T cells (2 × 10^5^ cells/well) were cotransfected with 2 µg of pRad18-Myc and 2 µg of pcDNA3-HA-L1 ORF1. The cell lysates were treated with or without 100 µg of RNase A (Nacalai tesque) and then immunoprecipitated with anti-Myc antibody, followed by immunoblotting analysis using either anti-Myc or anti-HA antibody. The results of Western blot analysis of 1/10 of the cellular lysates with anti-HA or anti-Myc-tag antibody are also shown. The experiment was replicated twice with similar results. (**C**) Endogenous Rad18 binds to L1 ORF1p. 293T cells (2 × 10^5^ cells/well) were transfected with either 4 µg of pJM101 or 4 µg of pcDNA3-L1 ORF1-HA. The cell lysates were immunoprecipitated with anti-Rad18 (A301-340A) antibody, followed by immunoblotting analysis using either anti-Rad18 or anti-hORF1p (SE-6798) antibody. The results of Western blot analysis of 1/10 of the cellular lysates with anti-Rad18 or anti-hORF1p antibodies are also shown. The experiment was replicated twice with similar results. (**D**) Subcellular localization of Rad18 and L1 ORF1p. 293T cells (2 × 10^4^ cells/well) cotransfected with 100 ng of Myc-tagged Rad18 (WT, ∆6BD, or ∆C2)-expressing plasmid and 100 ng of pcDNA3-HA-L1 ORF1 were examined by confocal laser scanning microscopy. Cells were stained with anti-HA (HA-7) and anti-Myc-tag mAb-Alexa Fluor 488 antibodies and then visualized with Alexa Fluor 594 (L1 ORF1p) or Alexa Fluor 488 (Rad18). Nuclei were stained with DAPI. The arrow indicates cytoplasmic foci. The colocalization frequency is shown. We observed twenty co-expressed cells.

**Figure 7:**
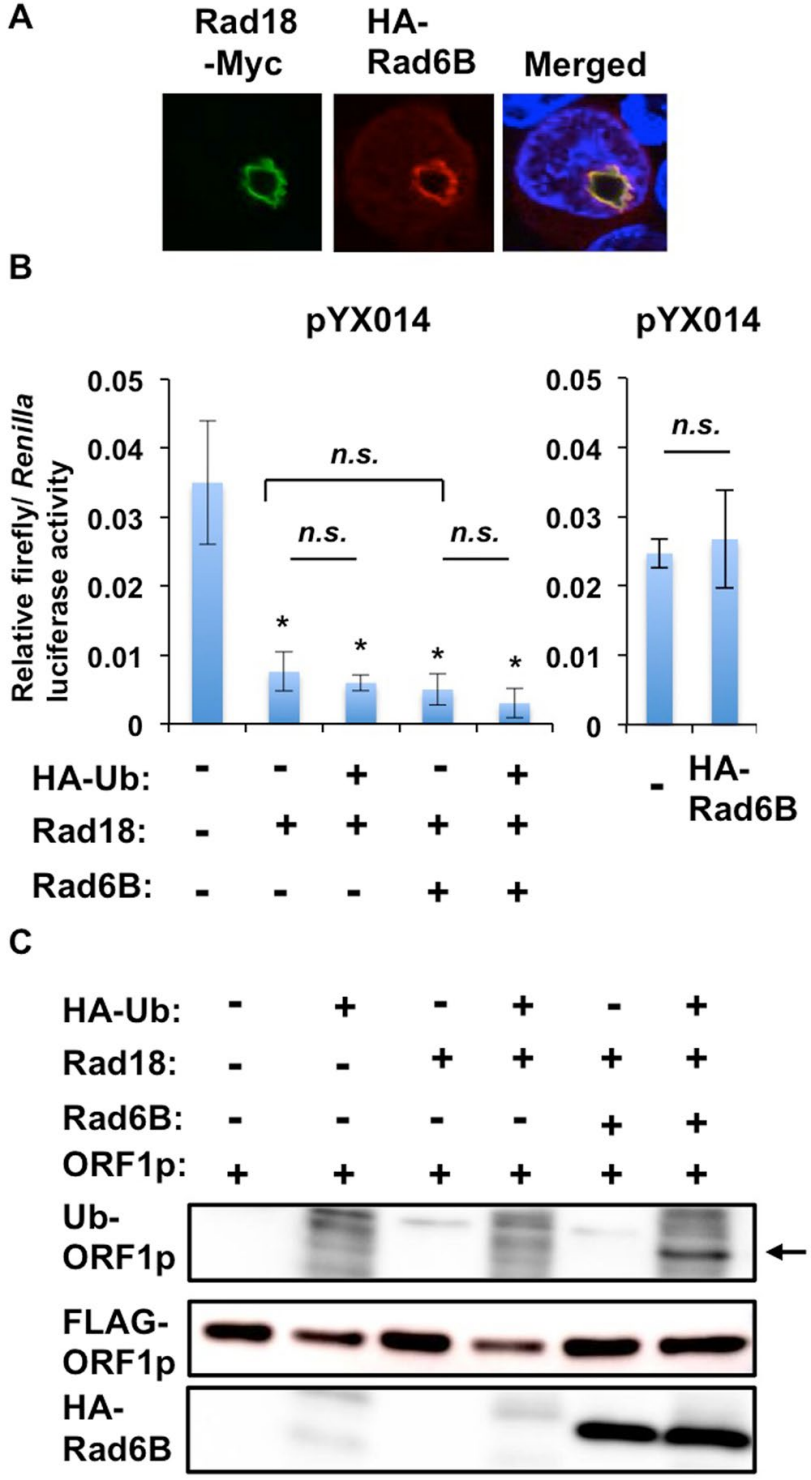
Effect of Rad6/Rad18 complex on L1 retrotransposition. (**A**) Subcellular localization of Rad18 and Rad6. 293T cells (2 × 10^4^ cells/well) cotransfected with 100 ng of Myc-tagged Rad18-expressing plasmid and 100 ng of pcDNA3-HA-Rad6B were examined by confocal laser scanning microscopy. Cells were stained with anti-HA (HA-7) and anti-Myc-tag mAb-Alexa Fluor 488 antibodies and then visualized with Alexa Fluor 594 (Rad6B) or Alexa Fluor 488 (Rad18). Nuclei were stained with DAPI. (**B**) 293T cells (2 × 10^4^ cells/well) were co-transfected with 100 ng of pYX014, 100 ng of pRad18-Myc, 100 ng of pcDNA3-HA-Rad6B, and/or 50 ng of pHA-Ub. Luciferase assays were performed three days after transfection in three independent experiments. Graph shows the mean (±SEM) firefly luciferase activity normalized with *Renilla* luciferase activity (*p < 0.05; *n.s*., not significant). (**C**) Detection of ubiquitinated L1 ORF1p. 293T cells (2 × 10^4^ cells/well) were co-transfected with 2 µg of pcDNA3-FLAG-L1 ORF1, 2 µg of pRad18-Myc, 2 µg of pcDNA3-HA-Rad6B, and/or 1 µg of pHA-Ub. Cells were cultured for 2 days, lysed, and subjected to Western blotting to detect ubiquitinated ORF1p using anti-HA antibody (3F10). Western blotting of the cell lysates with anti-FLAG, anti-HA, and anti-*ß*-actin antibodies is also shown, respectively.

To examine whether Rad18 binds to L1 ORF1p, the lysates of 293T cells co-expressing Myc-tagged Rad18 (WT, ∆6BD, or ∆C2) and HA-tagged L1 ORF1p were immunoprecipitated with anti-HA antibody, followed by Western blotting with anti-Myc-tag or anti-HA antibody. Consequently. WT Rad18 and the ΔC2 mutant were co-immunoprecipitated with HA-tagged L1 ORF1p (Fig. 6A), indicating that Rad18 binds to L1 ORF1p. However, the Δ6BD mutant failed to bind to L1 ORF1p (Fig. 6A). To further confirm this interaction, we examined the reciprocal immunoprecipitation analysis with either anti-Myc-tag or anti-Rad18 antibody. As expected, HA-tagged L1 ORF1p was co-immunoprecipitated with Myc-tagged Rad18 (Fig. 6B). Interestingly, RNase treatment could facilitate the interaction of L1 ORF1p with Rad18 (Fig. 6B), indicating that Rad18 interacts with L1 ORF1p in an RNA-independent manner. In this regard, RNA may compete with Rad18 for the ORF1p-binding, since L1 ORF1p is an RNA-binding protein. Furthermore, endogenous Rad18 co-immunoprecipitated with both pJM101-derived ORF1p and HA-tagged ORF1p using the lysates of 293T cells expressing ORF1p derived from pJM101 or HA-tagged ORF1p (Fig. 6C). We next examined their subcellular localization in 293T cells co-expressing Myc-tagged Rad18 (WT, ∆6BD, or ∆C2) and HA-tagged L1 ORF1p by using a confocal laser scanning microscopy (FV1200, Olympus). When either Rad18 or L1 ORF1p alone was expressed in 293T cells, we observed that WT and ∆C2 Rad18 predominantly localize into the nuclear foci with ring-like structure, while L1 ORF1p localizes in cytoplasmic foci such as stress granules or P-bodies. Notably, WT Rad18 and ∆C2 sequestered L1 ORF1p into the Rad18-nuclear foci from cytoplasmic foci, when both proteins were co-expressed (Figs 6D and S3). In contrast, the ∆6BD mutant showed the diffuse nuclear localization. In this context, the ∆6BD mutant failed to sequester L1 ORF1p in the nucleus (Fig. 6D). However, L1 ORF1p still localized in the cytoplasmic foci, even in the presence of the ∆6BD mutant (Fig. 6D, arrow). Thus, the Rad6-binding seems to be important for regulation of L1 mobility. As previously reported, Rad6, an E2 ubiquitin ligase, colocalized with Rad18 in Rad18-nuclear foci with ring-like structure (Fig. 7A). Over-expression of Rad6B alone did not affect L1 retrotransposition and we failed to observe the synergistic inhibition of L1 mobility when Rad6B and Rad18 were co-expressed even in the presence of ubiquitin (Ub) (Fig. 7B). However, we detected the ubiquitinated L1 ORF1p, when Rad18, Rad6, and HA-tagged Ub were co-expressed in 293T cells (Fig. 7C), indicating the Rad6-Rad18 complex ubiquitinates L1 ORF1p. Altogether, Rad18 seems to be required for regulation of L1 mobility.

## Discussion

L1 is a potent insertional mutagenic agent, which induces genomic instability, genetic disorders, and cancers^1^. In fact, insertion of L1 into the human genome can cause hemophilia or colon cancer^43,44^. To avoid such deleterious retrotransposition, host cells have evolved defense mechanisms protecting cells against L1 retrotransposition including epigenetic regulation through DNA methylation and host restriction factors. Indeed, Krüppel-associated box (KRAB)-containing zinc-finger protein (KRAB-ZFP/ZNF) and KRAB-associated protein 1 (Kap1, also known as TRIM28) are key determinants for transcriptional silencing of endogenous retroelements such as L1 and endogenous retroviruses^45–49^. In addition, APOBEC3, MOV10, and SAM domain and HD domain containing protein 1 (SAMHD1) restrict L1 retrotransposition^1,50–57^. These restriction factors were originally identified as HIV-1 restriction factors. In this study, we have demonstrated that Rad18 restricts L1 retrotransposition. Similarly, Rad18 also interacts with HIV-1 integrase and restricts HIV-1 infection^31,32^. On the other hand, HIV-1 integration and L1 insertion generates DSBs in the human genome, resulting in activation of host DNA damage sensors such as ATM kinase. Consequently, DNA repair proteins and DNA damage sensors have been implicated in L1 retrotransposition. Indeed, ATM and ERCC1/XPF complex suppresses L1 retrotransposition^15,19^, while NHEJ repair pathway such as Ku70 and DNA ligase IV is required for efficient L1 retrotransposition^16^ or restricts L1 retrotransposition^17,18^. Furthermore, Rad18 restricts L1 as well as Alu retrotransposition (Figs 1 and 3). Since L1 is required for the retrotransposition of Alu repeats in humans and SINE-VNTR-Alu (SVA)^40,41^, Rad18 seems to restrict Alu retrotransposition through the suppression of L1 mobility and may restrict SVA retrotransposition.

Rad18, an E3 ubiquitin ligase recruited to the DNA damage sites, induces monoubiquitination of PCNA, and contributes to DNA repair^25,26^. In this regard, PCNA interacts with ORF2p via a PIP box motif ^27^. Importantly, this interaction is critical for L1 retrotransposition^27^. Therefore, Rad18 may regulate L1 mobility through monoubiquitination of PCNA. Interestingly, we recently reported that a cell cycle regulator p21^Waf1^, a PCNA-interacting protein, restricts L1 mobility through an inhibition of L1 ORF2p reverse transcriptase activity^58^. Furthermore, Rad18 may recruit L1-generating DSB site in the human genome and ubiquitinate L1 protein, which leads to affecting L1 retrotransposition. In fact, the ∆6BD Rad18 mutant without ubiquitination activity, failed to suppress L1 retrotransposition (Fig. 4). Accordingly, we found that WT and ∆C2 Rad18 binds to L1 ORF1p, and sequesters L1 ORF1p into the Rad18-nuclear foci from cytoplasmic P-body or stress granule (Fig. 6D). In this context, WT and the ∆C2 mutant could strongly suppress L1 retrotransposition (Fig. 4). In contrast, ∆6BD, with a diffuse nuclear localization, did not sequester L1 ORF1p into the nuclear foci and failed to suppress L1 retrotransposition. Furthermore, we demonstrated the ubiquitinated L1 ORF1p when Rad18, Rad6 and Ub were co-expressed (Fig. 7). Thus, the ubiquitination activity of Rad18 might be important for the Rad18 restriction function for L1 mobility. However, we failed to observe the synergistic inhibition of L1 mobility when Rad6B and Rad18 were co-expressed even in the presence of ubiquitin (Ub) (Fig. 7B). This reason may be explained as follows: First, endogenous Rad6 is abundant (Fig. S2B) and enough for fully Rad18-mediated restriction of L1 mobility. Second, both Rad6A and Rad6B may be required for Rad18-mediated restriction of L1 mobility, since we only expressed Rad6B. Further experiments are needed to be clarified this issue.

Taken together, Rad18 seems to act as a guardian of the human genome against invading exogenous retroviruses such as HIV-1 and endogenous retroelements.

## Methods

### Cell culture

293T, HeLa and HCT116 cells were cultured in Dulbecco’s modified Eagle’s medium (DMEM; Life Technology, Carlsbad, CA, USA) with high glucose (4.5 g/L) supplemented with 10% fetal bovine serum (FBS) and 100 U/ml penicillin/streptomycin.

### Plasmid construction

To construct pcDNA3-HA-Rad6B, a DNA fragment encoding Rad6B was amplified from HuH-7, human hepatoma cell line, cDNA by PCR using KOD-Plus DNA polymerase (TOYOBO, Osaka, Japan) and the following pairs of primers: 5′-CGGGATCCATGTCGACCCCGGCCCGGAGGA-3′ (Forward), 5′-CCGGCGGCCGCTTATGAATCATTCCAGCTT-3′ (Reverse). To construct pcDNA3-FLAG-MOV10, a DNA fragment encoding MOV10 was amplified from HuH-7 cDNA by PCR using KOD-Plus DNA polymerase (TOYOBO, Osaka, Japan) and the following pairs of primers: 5′-CGGGATCCAAGATGCCCAGTAAGTTCAGCTG-3′ (Forward), 5′-CCGCTCGAGTCAGAGCTCATTCCTCCACTCTG-3′ (Reverse). The obtained DNA fragments were subcloned into either the *Bam*HI-*Not*I sites of the pcDNA3-HA vector (Rad6B) or the *Bam*HI-*Xho*I sites of the pcDNA3-FLAG vector (MOV10), and the nucleotide sequences were determined by Sanger sequencing.

### Western blot analysis

Cells were lysed in buffer containing 50 mM Tris-HCl (pH 8.0), 150 mM NaCl, 4 mM EDTA, 1% Nonidet (N) P-40, 0.1% sodium dodecyl sulfate (SDS), 1 mM dithiothreitol (DTT), and 1 mM phenylmethylsulfonyl fluoride (PMSF). Supernatants from these lysates were subjected to SDS-polyacrylamide gel electrophoresis, followed by immunoblot analysis using anti-Rad18 (A301-340A; Bethyl Lab, Inc., Montgomery, TX, USA), anti-*ß*-actin (A5441; Sigma, Saint Louis, MI, USA), anti-HA (HA-7; Sigma), anti-Myc-tag mAb (PL-14; MBL, Nagoya, Japan) or anti-hORF1p (SE-6798)^34^ as primary antibodies. We used peroxidase-conjugated Donkey anti-Rabbit IgG (H + L) (Jackson ImmunoResearch) or Amersham ECL peroxidase-linked Sheep anti-Mouse IgG (GE Healthcare) as secondary antibodies. The proteins were detected by using Western Lightning Plus ECL, enhanced chemiluminescence substrate (PerkinElmer, Waltman, MA) and Amersham Imager 600 (GE Healthcare Bio-Sciences, Uppsala, Sweden).

### Luciferase assay

Plasmids were transfected into 293T cells (2 × 10^4^ cells) using TransIT-LT1 transfection reagent (Mirus Bio LLC, Madison, WI, USA). Luciferase assays were performed 72 h after transfection using luciferase assay reagent according to the manufacturer’s instructions (Promega, Madison, WI, USA). All transfections utilized the same amount of total plasmid DNA owing to the addition of empty vector to make up the balance in transfection mixtures. Results were obtained through three independent transfections. A lumat LB9507 luminometer (Berthold, Bad Wildbad, Germany) was used to detect the luciferase activity. The pYX014 plasmid is a dual-luciferase reporter, in which firefly luciferase is used as the retrotransposition indicator and *Renilla* luciferase is encoded on the same plasmid for normalization^33^.

### RNA interference

Oligonucleotides with the following sense and antisense sequences were used for the cloning of short hairpin RNA (shRNA)-encoding sequences targeted to Rad18 in a lentiviral vector: 5′- GATCCCCGCAGTTTGCTTTAGAGTCATTCAAGAGATGACTCTAAAGCAAACTGCTTTTTGGAAA-3′ (sense), 5′-AGCTTTTCCAAAAAGCAGTTTGCTTTAGAGTCATCTCTTGAATGACTCTAAAGCAAACTGCGGG-3′ (antisense). The oligonucleotides above were annealed and subcloned into the *Bgl*II-*Hin*dIII sites, downstream from an RNA polymerase III promoter of pSUPER^59^, to generate pSUPER-Rad18i. To construct pLV-Rad18i, the *Bam*HI-*Sal*I fragments of the pSUPER-Rad18i were subcloned into the *Bam*HI-*Sal*I sites of pRDI292, an HIV-1-derived self-inactivating lentiviral vector containing a puromycin resistance marker allowing for the selection of transduced cells^60^.

### Lentiviral vector production

The vesicular stomatitis virus (VSV)-G-pseudotyped HIV-1-based vector system has been described previously^61,62^. The lentiviral vector particles were produced by transient transfection of 293T cells with the second-generation packaging construct, pCMV∆R8.74, and the VSV-G-envelope-expressing plasmid, pMD2G^61,62^, as well as pLV Rad18i or pLVshCon lentiviral vector with TransIT-LT1 transfection reagent.

### Real-time RT-PCR

Total RNA was isolated using RNeasy Mini kit (Qiagen) and treated with Turbo DNA-free kit (Life Technology). The total RNA was reverse transcribed with oligo(dT)12-18 primer (Life Technology). The quantitative RT-PCR analysis for Rad18 and *ß*-*actin* mRNA was performed by real-time LightCycler PCR (Roche). We used the following forward and reverse primer sets: Rad18, TCTGTATGCATGGGACAGGA (Forward), TCAGGTTCCAATTCCTCTGG (Reverse); *ß*-*actin*, TGACGGGGTCACCCACACTG (forward), AAGCTGTAGCCGCGCTCGGT (reverse).

### Immunoprecipitation

Cells were lysed in buffer containing 10 mM Tris-HCl (pH 8.0), 150 mM NaCl, 1 mM EDTA, 0.1% NP-40, 10 mM NaF, 1 mM DTT and 1 mM PMSF. Lysates were pre-cleared with 30 µl of protein-G-Sepharose (GE). Pre-cleared supernatants were incubated with 5 µg of anti-HA antibody (3F10; Roche Diagnostics, Mannheim, Germany) or anti-Myc-tag mAb (PL-14; MBL) at 4 °C for 1 h. Following absorption reaction of the precipitates with 30 µl of protein-G-Sepharose resin for 1 h, the resin was washed four times with 700 µl of lysis buffer. Proteins were eluted by boiling the resin for 5 min in 2X Laemmli sample buffer. The proteins were then subjected to SDS-PAGE, followed by immunoblotting analysis using either anti-HA (HA-7; Sigma) or anti-Myc-tag mAb antibody (PL-14; MBL).

### Immunofluorescence and confocal microscopy analysis

293T cells were grown on Lab-Tek 2 well chamber slide (Nunc, Thermo) at 2 × 10^4^ cells per well and transfected the next days using TransIT-LT1 transfection reagent. Two days after transfection, the cells were fixed in 3.6% formaldehyde in 1X phosphate-buffered saline (PBS), permeabilized in 0.1% NP-40 in 1X PBS at room temperature, and incubated with anti-HA antibody (HA-7) at a 1:300 dilution in 1X PBS containing 3% bovine serum albumin (BSA) at 37 °C for 30 min. We also used anti-DDX6 (A300-460A; Bethyl), anti-FLAG (M2; Sigma), anti-G3BP1 (A302-033A; Bethyl) or anti-PABP (ab21060; Abcam) antibodies as primary antibodies. Cells were then stained with Donkey anti-mouse IgG (H + L) secondary antibody, Alexa Fluor 594 conjugate (Thermo Fisher Scientific Inc., Waltham, MA, USA), followed by staining with anti-Myc-tag mAb-Alexa Fluor 488 (M047-A48; MBL) at a 1:300 dilution in PBS containing BSA at 37 °C for 30 min. Nuclei were stained with DAPI (4′, 6′-diamidino-2-phenylindole). Following washing 3 times in 1X PBS, the coverslides were mounted on slides using SlowFade Gold antifade reagent (Life Technology). Samples were analyzed under a confocal laser-scanning microscope (FV1200; Olympus, Tokyo, Japan).

### L1 retrotransposition assay

The L1 retrotransposition assay was performed as follows: HeLa cells (2 × 10^5^ cells/well in six- well plates) were co-transfected with 2 µg of pJM101^35–37^ and 2 µg of pRad18-Myc^22^. Two days after transfection, cells were seeded into 100 mm culture dishes and selected with G418 (Promega) at 0.6 mg/ml for three weeks. G418-resistant colonies were fixed and stained with 0.6% Coomassie brilliant blue in 50% methanol and 10% acetate. The Number of colonies was counted by Eliphoto counter (Minerva Tech, Tokyo, Japan).

### Alu retrotransposition assay

The L1-deriven Alu retrotransposition assay was performed as previously described^41^. HeLa cells (2 × 10^5^ cells/well in six-well plates) were co-transfected with 2 µg of pAlu pA + neoTET^39^, 2 µg of pJM101 L1-RP∆Neo^36,41^ and 2 µg of pRad18-Myc^22^. Two days after transfection, cells were seeded into 100 mm culture dishes and selected with G418 (Promega) at 0.6 mg/ml for four weeks. G418-resistant colonies were fixed and stained with 0.6% Coomassie brilliant blue in 50% methanol and 10% acetate. The Number of colonies was counted by Eliphoto counter (Minerva Tech, Tokyo, Japan).

### Statistical analysis

The statistical significance of the inter-sample differences was determined using the paired Student’s *t* test. *p* values of < 0.05 were considered statistically significant.

## Electronic supplementary material

Supplementary Information
