# Supplementary material for: DNA repair protein Rad18 restricts LINE-1 mobility
Source: Sci Rep. 2018 Oct 26;8:15894. doi: 10.1038/s41598-018-34288-9 (PMC6203705; doi:10.1038/s41598-018-34288-9)
Supplement: Supplementary file 1 — Supplementary Information [file 41598_2018_34288_MOESM1_ESM.pdf]

Title: DNA Repair Protein Rad18 restricts LINE-1 mobility

Authors: Yasuo Ariumi, Koudai Kawano, Mariko Yasuda-Inoue, Misao Kuroki, Hiroyuki Fukuda, Rokeya Siddiqui, Priscilla Turelli & Satoshi Tateishi

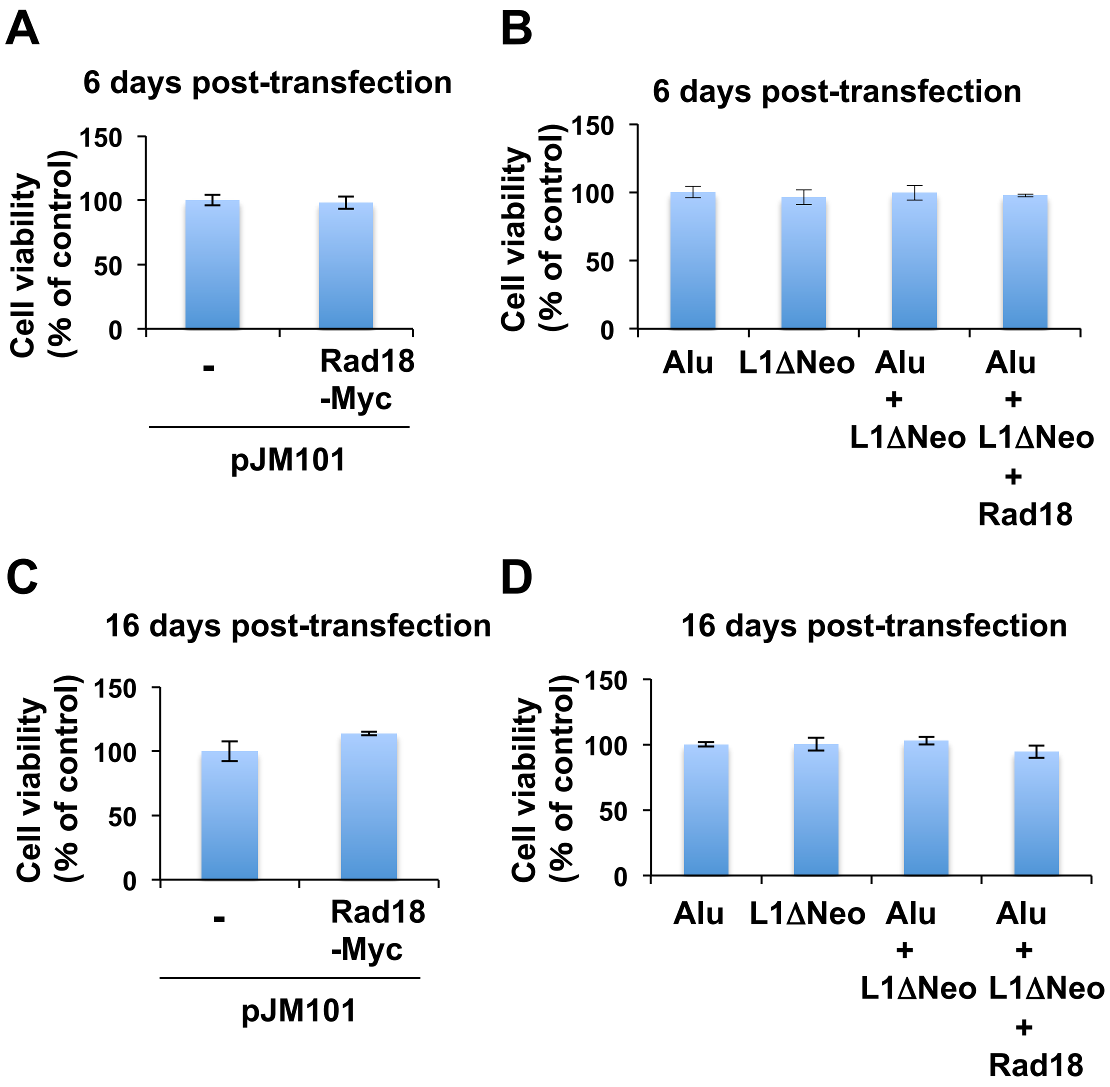

Supplementary Fig. S1

Effect of Rad18 on cell growth and viability. **(A, B)** HeLa cells (2×10^5 cells) were co-transfected with 2 μg of pJM101 and/or 2 μg of pRad18-Myc **(A)**, or 2 μg of pAlu pA+neoTET, 2 μg of pJM101 L1-RP Δneo and/or 2 μg of pRad18-Myc **(B)**. Next day, the cells (5×10^3 cells/well) were plated onto 96-well plates and cultured for 5 days. **(C, D)** HeLa cells (2×10^5 cells) were co-transfected with 2 μg of pJM101 and/or 2 μg of pRad18-Myc **(C)**, or 2 μg of pAlu pA+neoTET, 2 μg of pJM101 L1-RP Δneo and/or 2 μg of pRad18-Myc **(D)**. After the cells were cultured for 15 days, the cells (5×10^3 cells/well) were plated onto 96-well plates and cultured for 1day. The cells were subjected to the colorimetric 3-(4,5-dimethylthiazol-2-yl)-2,5-diphenyltetrazolium bromide (MTT) assay according to the manufacturer's instructions (Cell Proliferation Kit I; Roche). The absorbance was read using a microplate reader (xMark microplate spectrophotometer; Bio-rad) at 550 nm with a reference wavelength of 640 nm. The results shown are means from three independent experiments. Error bars indicate standard deviations.

Fig.S1

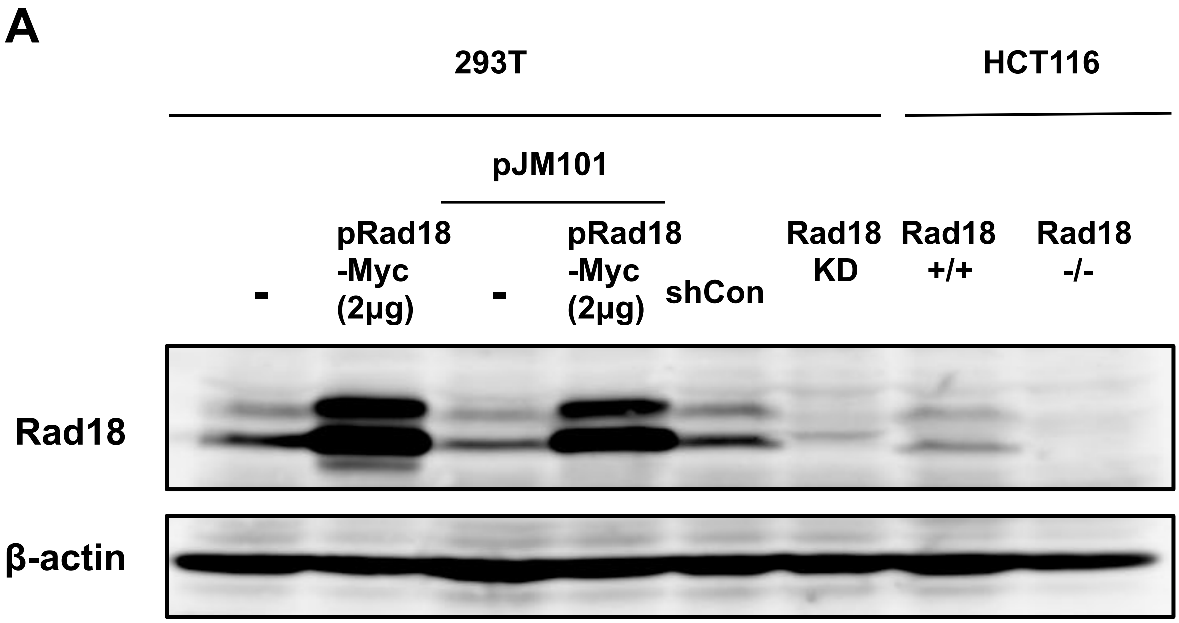

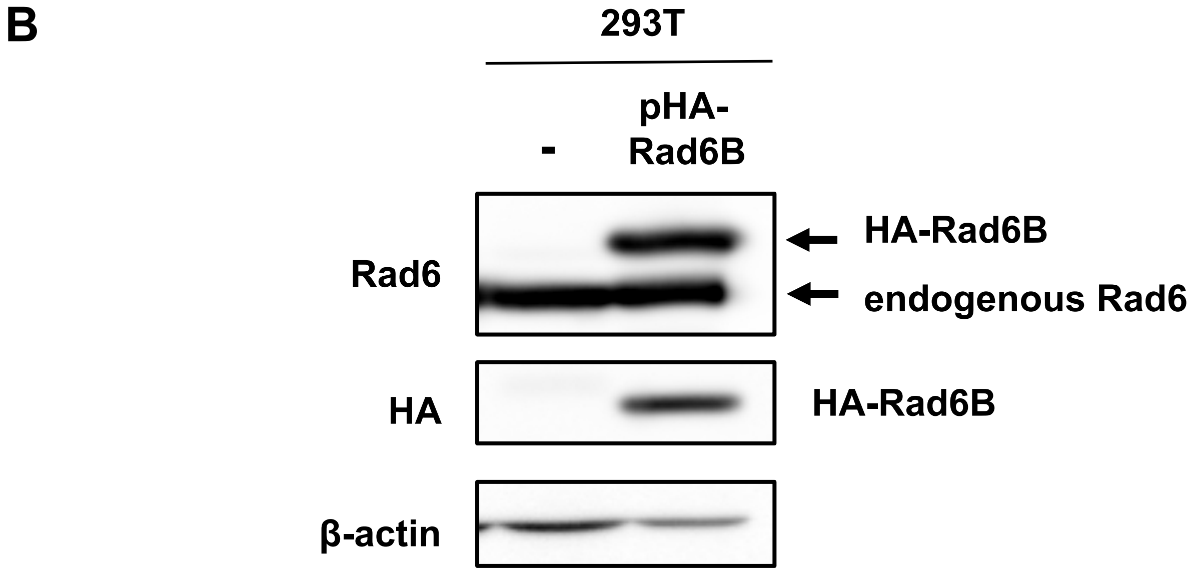

Supplementary Fig. S2

(A) The levels of Rad18 protein expression were analyzed by Western blotting using the cell lysates of 293T cells, 293T cells trasfeced with 2 μg of pRad18-Myc, 293T cells co-transfected with 2 μg of pJM101 and/or 2 μg of pRad18-Myc, Rad18-knockdown 293T (Rad18KD) or the control cells (shCon), Rad18$^{+/+}$ or Rad18$^{-/-}$ HCT116 cell. The results of Western blot analysis with anti-Rad18 or anti-β-actin antibody are shown.

(B) The levels of endogenous Rad6 and exogenous HA-Rad6B protein expression were analyzed by Western blotting using the cell lysates of 293T cells trasfeced with 2 μg of pcDNA3-HA or pcDNA3-HA-Rad6B. The results of Western blot analysis with anti-Rad6, anti-HA, or anti-β-actin antibody are shown.

Fig.S2

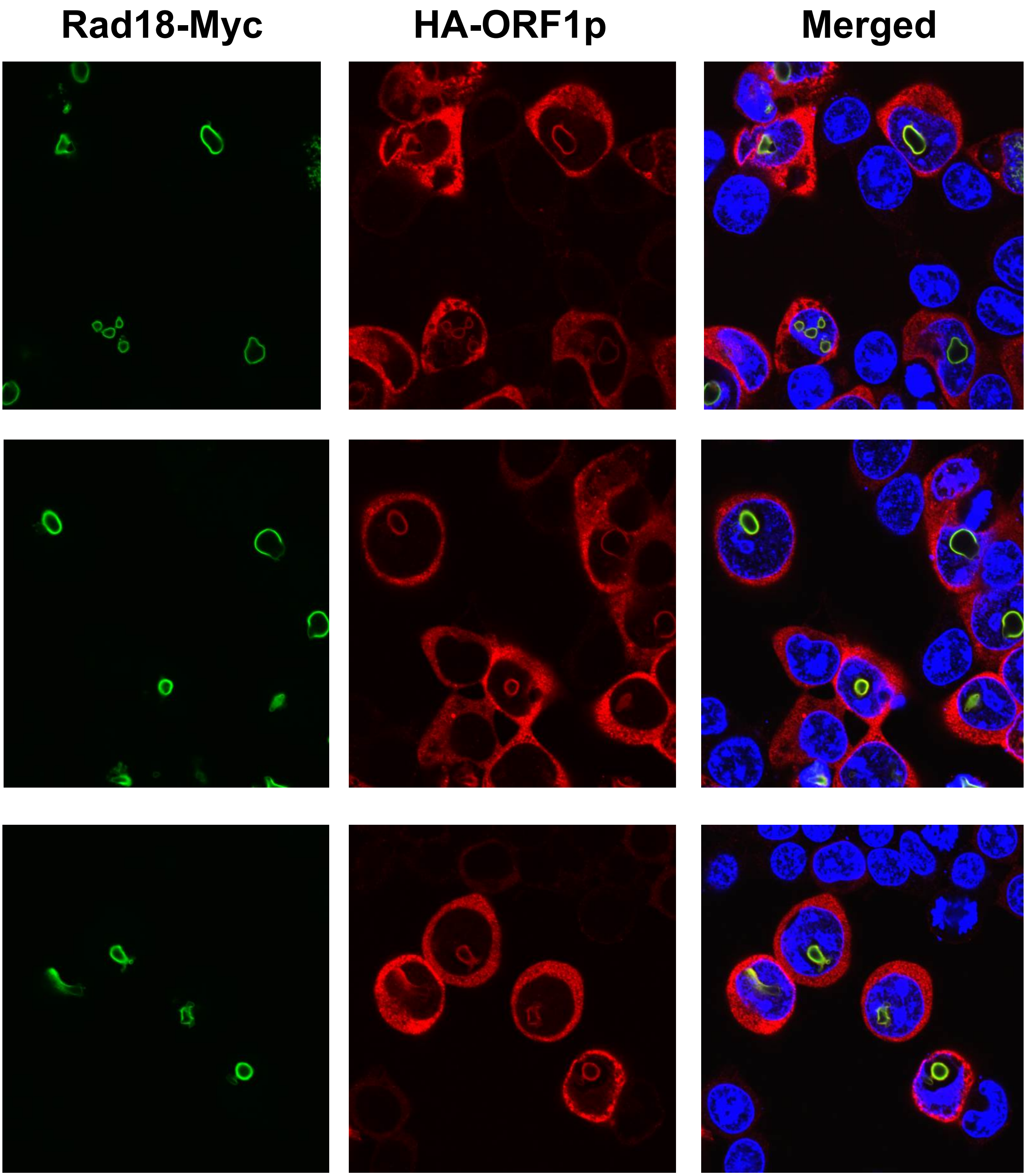

Supplementary Fig. S3

Subcellular localization of Rad18 and L1 ORF1p. 293T cells (2×10^4 cells/well) cotransfected with 100 ng of Myc-tagged Rad18-expressing plasmid and 100 ng of pcDNA3-HA-L1 ORF1 were examined by confocal laser scanning microscopy. Cells were stained with anti-HA (HA-7) and anti-Myc-tag mAb-Alexa Fluor 488 antibodies and then visualized with Alexa Fluor 594 (L1 ORF1p) or Alexa Fluor 488 (Rad18). Nuclei were stained with DAPI.

Fig.S3
